# For Whom the Note Scrolls: A Brief History of the Medical Record's Role in Education and the Risks It Faces in the Age of OpenNotes

**DOI:** 10.1212/NE9.0000000000200147

**Published:** 2024-09-09

**Authors:** Adina Wise

**Affiliations:** From Mount Sinai Beth Israel, Icahn School of Medicine at Mount Sinai, New York, NY.

## Abstract

Dating back to ancient civilizations when records were carefully transcribed onto papyrus, clinical documentation has long served as a cornerstone of medical—and especially neurologic—education. From the case histories of Hippocrates to the diurnal patient logs used by trainees in the 18th and 19th centuries, clinical notes have an extended history as invaluable instruments of pedagogy, scholarly practice, and interprofessional communication. The novel paradigm introduced by Lawrence Weed in the 1950s, advocating for the problem-oriented medical record system, revolutionized the clinical note template and emphasized the need for physicians' carefully considered analyses of a patient's presentation to be clearly reflected in well-organized documentation. In the realm of medical records today, however, a profound shift is underway, largely propelled by the emergence of electronic medical records, the OpenNotes mandate of the federal 21st Century Cures Act, and, most recently, artificial intelligence (AI). Appropriately, patients now have full access to their medical records, but this raises critical questions. Should clinical notes now prioritize patient comprehension over their traditional role as educational instruments, *aide-mémoire*, and repositories of detailed assessments and insights? What role, if any, should AI have in the creation of physician notes and patient-facing clinical documents? These tensions underscore the delicate balance between transparency and the preservation of notes' clinical integrity and analytical depth. As we navigate the path forward, finding an equilibrium between openness and the continued utility of medical records as tools for education and professional communication will be imperative.

## Introduction

Clinical documentation is integral to the practice of neurology, serving as a cornerstone of clinical reasoning, education, and effective patient care. For the past 50 years, medical students have been taught that the best clinical notes synthesize all the gathered information and develop a well-considered, focused list of problems, alongside an analysis of plausible explanations, followed by clear and concise treatment or workup recommendations.^1^ However, the medical record was not always beholden to a precise template or role. Rather, since early antiquity—alongside advances in scientific knowledge, procedural acumen, and pedagogical methods—clinical documentation has perennially evolved. Over the past few years, the widespread implementation of patient note-sharing policies and, more recently, the rapid infiltration of artificial intelligence (AI) systems have introduced new challenges and complexities into the way physicians approach clinical documentation. Reflecting on the history of the medical record provides an essential context for today's uncertainties and illuminates the necessity of carefully considering our path forward.

## Historical Overview of Medical Recordkeeping

The earliest examples of clinical documentation still in existence took the form of case series, which were transcribed onto papyrus in Ancient Egypt and date back to approximately 2000 BC.^2^ At that time, medicine was still in its infancy, and conjured etiologies were replete with fairly equal measures of magic and reason.^2,3^ These texts thus served as didactic material that could be used to teach apprentices and guide the management of future patients^4,5^ because tracking the daily vicissitudes of a sick individual's condition was, at this point, much less of a priority. Even so, and to a surprising degree, the templates of these archaic, hieroglyphic texts resemble modern patient charts: they begin by describing the relevant examination methods, proceed to lay out the pertinent diagnoses, and conclude with recommended remedies.^6,7^

In the time of Hippocrates, from the late fifth to early fourth century BC, medical recordkeeping expanded significantly and case documents from this era are especially remarkable for their newfound focus on chronology.^5^ By zeroing in on the precise order in which symptoms developed and the progression of undertaken attempts to heal, physicians were able to start drawing conclusions about the relationship between ailments, treatments, and outcomes. This methodology was, similarly, of utmost importance to medical education. Through careful surveillance and examination, with corresponding documentation, Hippocrates and his disciples were the first physicians to invite true clinical reasoning into the practice of neurology and medicine.^8,9^

Notwithstanding these ever-more sophisticated forms of recordkeeping, it was not until the 18th and 19th centuries that updating and maintaining diurnal patient logs became the norm.^10,11^ As formal hospital-based training programs for physicians and surgeons were introduced across Europe, the careful composition of daily case histories gained traction as means of teaching and scholarly practice.^11,12^ At the Charité Hospital in Berlin, for example, the recording of patient examinations and diet orders, which began as a purely administrative endeavor, evolved into an essential component of the training curriculum as the educational value of preparing and updating records was realized and began to fuse with the clerical necessity of their maintenance.^12^ In Scotland, meanwhile, medical students were bestowed with the much desired opportunity to access, study, and copy records that had been composed by senior doctors—an implied acknowledgment of this activity's academic utility.^13^ Physicians in training at the Edinburgh Hospital were also encouraged to carefully observe and record their own detailed patient assessments and to apply these toward the advancement of the existing medical canon.^13^ More broadly, written notes became useful for junior doctors who were required to present the details of a patient's history to senior physicians, thus propagating an established tradition of revealing one's medical acumen through the skilled retelling of illness histories.^11,14^ Finally, and no less importantly, bedside charts that were updated regularly could now serve as a method of communication and *aide-mémoire* for all members of the medical team.^11,15^ Even so, what information was to be included in these notes, and how, remained ambiguous—and varied greatly between practitioners and institutions—for more than a 100 years.

## Introduction of the Problem-Oriented Medical Record

It was in the 1950s that Dr. Lawrence Weed, then a professor of medicine and pharmacology at Yale University, became concerned about the haphazard way that his residents were collecting and documenting patient data. Their “chaotic” approach to writing notes, he felt, was a microcosm of a systemic malady, one which was negatively affecting how physicians thought, how trainees learned, and, ultimately, how patients were cared for.^1,16^ In a number of published editorials from the 1960s, Weed, therefore, made impassioned arguments for the introduction of a problem-oriented medical record.^16-18^

A critical aspect of Weed's^16^ philosophy was that the medical record should serve as an educational tool requiring that the student go “through the labor of organizing his thoughts” and as a means of “communication for all time” between physicians and other members of the health care team. To a large extent, medical records had been serving these purposes, however heterogeneously, since the time of Charcot, Virchow, and Osler. However, Weed^16^ insisted that the task follow a prescribed template—our modern Subjective, Objective, Assessment, Plan (SOAP) note—and be consistently performed with a high degree of intellectual attentiveness and rigor.

Perhaps a testament to the dire need for such reforms, Weed's system caught on and, over time, spread throughout the country and the world.

## Patient Note-Sharing and OpenNotes

Although Weed's insistence on maintaining clinical notes that exemplify “accuracy, uniformity, precision, and completeness” was explicitly in the interest of improved patient care, his extensive writings on this subject never directly mention a situation in which the patient themselves might interact with their records.^16-18^ This fact is indeed somewhat surprising because it was in the latter decades of the 20th century that a highly paternalistic model of doctoring began to be replaced by a patient-physician relationship framework that favored individual autonomy and shared decision making.^19^

Exemplifying this shift, a mere 5 years after Weed published his 2-part treatise, “Medical Records that Guide and Teach,” Budd Shenkin and David Warner wrote an editorial—“Giving the Patient his Medical Record: A Proposal to Improve the System”—which appeared within the pages of the very same well-respected journal.^20^ Therein, the authors argue that allowing patients unfettered access to their charts might enhance patient education, improve the quality of information in the records, and promote greater continuity of care.^20^

At the time, Shenkin and Warner's recommendations were met with some pushback, but they were not wrong. Over the past decade, several pilot note-sharing programs have been introduced and, in April of 2021, the OpenNotes provision of the federal 21st Century Cures Act went into effect, thus mandating fast and easy access to all components of one's medical record. The benefits of OpenNotes for patients have now been empirically explored and include improved memory and understanding of treatment plans, increased likelihood of taking medications, a sense of empowerment regarding medical care, and higher levels of trust in physicians and the health care team.^21-24^ Notwithstanding the unequivocal benefits of note-sharing for patients, however, challenges remain.

## Selecting an Audience: A Challenge Inherent to Patient Note-Sharing

A unique recent study of physician clinical documentation in the age of OpenNotes generated simple and complex versions of the same radiation oncology records and provided these to participants for review. While the simple notes were deemed easier to understand, both types performed poorly on objective tests of usability, cognitive workload, and participant satisfaction, leading study authors to conclude that “to make the notes useful, there is a need for dramatic improvements to the usability and content of the notes.”^25^

However, as exemplified by this determination, the easy accessibility of clinical notes has introduced a question that Shenkin and Warner, among others, did not anticipate: *For whom* is it most imperative that clinical notes be of use? Does the fact that the medical record is now readily available to patients necessitate that it be composed exclusively with that audience in mind? And, if we do pivot to crafting notes solely for the eyes of our patients (as some physicians already have), what complexities, critical details, and carefully considered assessments will we be leaving behind?

Several studies suggest that the way doctors compose notes is already changing.^26^ In a Swedish survey study, sizable cohorts of oncologists confessed that they had altered the way they write about obesity, addiction, mental health, and cancer while two-thirds (67.7%) admitted that they were more restrictive regarding what they include in notes overall.^27^ In a survey study of dermatopathologists in the United States, 18% reported that patient access to notes would lead them to change the way they describe lesions suspicious for cancer.^28^ Meaningful percentages of mental health physicians in both Sweden^29^ and the United States^30^ have also admitted to a more constrained approach in response to notes becoming available to patients, and specifically regarding what is written about certain diagnoses. A separate, large survey study of US clinicians also found that doctors (21% of specialists and 26% of primary care providers) had changed the way they document differentials while 22% of all physician respondents felt that the value of their notes for other clinicians had been diminished by note-sharing policies.^22^

Because these data points are based on survey responses and not direct analyses of note content, it is impossible to know the full extent and impact of the differences in note composition that have been reported. Even so, with more than 1 in 5 physicians endorsing a depreciation in their notes' interprofessional merit, it would seem that the question of how, and for whom, clinical notes should be written is well worthy of consideration.

The impetus for physicians' making changes to their documentation practices in the wake of OpenNotes is, almost always, a concern for the patient's emotional well-being.^27-29^ Doctors worry that their patients may be confused or anxious because of something they have written; thus, they decide to write cryptically, rephrase, abridge, or self-censor (it should go without saying that the removal of derogatory language or statements critical of the patient is imperative, and such verbiage should never have been there in the first place). Pertinently, physician disquietude around patient note-sharing may be nowhere more relevant than the field of neurology, where uncertainties abound and certain disease states may have a direct impact on a patient's ability to understand the medical provider's assessment of their condition.^e1^

However, if clinical notes are to be simplified with new readers in mind, what should happen to the potentially valuable, if more esoteric, content that has been redacted? Should we plainly assume that these innards were, in fact, extraneous and irrelevant, regarding physician training, crucial communication between providers, and capturing a physician's carefully organized process of thought?

Understanding the history of the medical record allows for the recognition that, in a post-OpenNotes world, a single document is being asked to work way beyond its duty-hour limits. If clinical notes are invaluable tools for physician education, contemplation, and communication and deserve to be maintained as such, then it may be infeasible for these dossiers to be ideally suited for patient consumption at the very same time. While patients should certainly have access to the entirety of their records, lay summaries, streamlined explanations, and other supplementary materials may need to exist as separate entries, ones that are no less readily available, within the chart.

However, physicians are already inundated with administrative tasks and overburdened by the electronic medical record's (EMR) insistence on interminable clicks in the interest of optimal billing rather than improved patient care.^e2^ If the OpenNotes mandate indirectly demands the creation of independent documents with disparate audiences in mind, we must consider how these would be composed and at whose feet this additional responsibility would fall.

## Future Directions: Avoiding the Perils, Embracing the Promise of AI

The recent advent of Health Insurance Portability and Accountability Act–compliant natural language processing (NLP) systems has the potential to meaningfully alter the physician's overgrown clerical terrain by taking on tasks which might otherwise detract from educational and care-based ventures.^e3,e4^ Although AI systems remain highly imperfect, if used appropriately, they could shift the onus of delivering patient-ready documentation away from practicing physicians and toward NLP. Epic, the EMR software company, is currently engaged in efforts to incorporate GPT functions onto its platform, and existing research has already shown that NLP may be capable of composing empathic and well-received patient communications.^e5,e6^ In an effort to ensure quality control, one could imagine a scenario in which the NLP's overall work product might be overseen by a patient education specialist or other, nonphysician team member, at least initially. This model would, ideally, allow students and teachers alike to focus on writing notes in the way that Lawrence Weed intended: with a focus on clinical reasoning and an orientation toward addressing the patient's medical problems.^e7,e8^

Even so, we must proceed with caution. AI systems have already begun to infiltrate the clinical workspace, and new studies reporting on their ability to all-but-entirely take over note-writing tasks are arriving on PubMed Central expeditiously.^e9^ However, for these tools to be used effectively, it is also imperative that medical students, residents, fellows, and practicing physicians receive guidance on how to use them—and how not to.^e10^ Scholarly journals have been swift to delineate rules and requirements pertaining to the use and limitations of AI in research articles. Similarly, the development of best practice recommendations should be undertaken by stakeholders in curriculum development and professional conduct pertaining to the use of NLP in clinical documentation, while taking into account ethical, legal, and educational priorities and the highly relevant history, evolution, and purpose of the medical record.

While NLP may, eventually, excel at documenting the history of present illness, medication list, social history, and imaging data, we should, perhaps, discourage AI's usurpation of the note's “Assessment and Plan,” wherein lies an invaluable opportunity for physicians to engage in reflective contemplation and critical thinking. With the same goal in mind, medical educators in neurology and beyond should, moving forward, consider an increased emphasis on a pedagogical format that has withstood the test of time: the case report (or case history). As Walter Bradford Cannon, a physician at Harvard, wrote in 1900, “the most important aspect of the case system is…. its great value in drilling the mind of the student to meet intelligently the difficulties of practice.”^e11^ If AI and note-sharing policies do dampen trainees' prospects for in-depth consideration of their patients' conditions, then the challenge of constructing extended narrative reports, detailing presentation, progression, and disposition, in combination with a review of the existing literature and one's own, novel ideas regarding pathology and treatment may provide at least a partial remedy. These dossiers would not need to be written with publication in mind (although this may be a welcome outcome) and could be assigned for completion during less work-intensive rotations, so as not to saddle exhausted residents with additional, unreasonable burdens. The aim of these exercises would, of course, be to maintain the most valuable educational elements of clinical documentation, just as the eminent physician and writer, Christoph Wilhelm Hufeland (1762–1836), so poignantly described:

When the noises of the day have died down and the stillness of the evening invites to quiet reflection, [the physician] should devote a few hours to calm contemplation of his patients….Here, in the still of the night, matters may appear in a different light; ideas and inspirations will arise in him that were impossible with the distractions of the day. It is only now, when the inner life awakens, that these impressions can find entry to the inner life and meet with genuine interest and attention. (As quoted in Hess 2016)^14^

## Conclusion

The balance between patient accessibility and the preservation of clinical documentation as a tool for medical training and interprofessional communication is delicate. As we look ahead, we must consider how best to evolve our practices to enhance patient understanding and involvement without sacrificing the careful cogitation and analysis—and documentation thereof—that are fundamental to the art of providing exceptional medical care.

## Appendix Author

**Table:**

Name	Location	Contribution
Adina Wise, MD	Mount Sinai Beth Israel, Icahn School of Medicine at Mount Sinai, New York, NY	Drafting/revision of the manuscript for content, including medical writing for content
